# Supplementary material for: Unveiling the Bioactive Potential of the Invasive Jellyfish Phyllorhiza punctata Through Integrative Transcriptomic and Proteomic Analyses
Source: Biomolecules. 2025 Aug 4;15(8):1121. doi: 10.3390/biom15081121 (PMC12383608; doi:10.3390/biom15081121)
Supplement: Supplementary file 1 [file biomolecules-15-01121-s001.zip › Suplementary Material/FileS3/PEPFOLD4/XP_065062106_8080/clusters.html]

 PEP-FOLD clusters


Download archive of all models  here   

|  |  |  |
| --- | --- | --- |
| # | file | sOPEP |
| # cluster 1 - Size: 5 |  |  |
|  | XP\_065062106\_8080-model1 | -7.44448 |
|  | XP\_065062106\_8080-model1.1 | -6.87866 |
|  | XP\_065062106\_8080-model1.2 | -6.85585 |
|  | XP\_065062106\_8080-model1.3 | -6.29948 |
|  | XP\_065062106\_8080-model1.4 | -6.22093 |
| # cluster 2 - Size: 44 |  |  |
|  | XP\_065062106\_8080-model2 | -7.1664 |
|  | XP\_065062106\_8080-model2.1 | -7.0749 |
|  | XP\_065062106\_8080-model2.2 | -7.0749 |
|  | XP\_065062106\_8080-model2.3 | -6.91455 |
|  | XP\_065062106\_8080-model2.4 | -6.7329 |
|  | XP\_065062106\_8080-model2.5 | -6.7329 |
|  | XP\_065062106\_8080-model2.6 | -6.66974 |
|  | XP\_065062106\_8080-model2.7 | -6.64385 |
|  | XP\_065062106\_8080-model2.8 | -6.64385 |
|  | XP\_065062106\_8080-model2.9 | -6.64385 |
|  | XP\_065062106\_8080-model2.10 | -6.63012 |
|  | XP\_065062106\_8080-model2.11 | -6.63012 |
|  | XP\_065062106\_8080-model2.12 | -6.63012 |
|  | XP\_065062106\_8080-model2.13 | -6.63012 |
|  | XP\_065062106\_8080-model2.14 | -6.56613 |
|  | XP\_065062106\_8080-model2.15 | -6.49993 |
|  | XP\_065062106\_8080-model2.16 | -6.49141 |
|  | XP\_065062106\_8080-model2.17 | -6.40248 |
|  | XP\_065062106\_8080-model2.18 | -6.40248 |
|  | XP\_065062106\_8080-model2.19 | -6.40248 |
|  | XP\_065062106\_8080-model2.20 | -6.29471 |
|  | XP\_065062106\_8080-model2.21 | -6.25326 |
|  | XP\_065062106\_8080-model2.22 | -6.25326 |
|  | XP\_065062106\_8080-model2.23 | -6.07258 |
|  | XP\_065062106\_8080-model2.24 | -6.07258 |
|  | XP\_065062106\_8080-model2.25 | -6.07258 |
|  | XP\_065062106\_8080-model2.26 | -6.0083 |
|  | XP\_065062106\_8080-model2.27 | -5.946 |
|  | XP\_065062106\_8080-model2.28 | -5.87436 |
|  | XP\_065062106\_8080-model2.29 | -5.77133 |
|  | XP\_065062106\_8080-model2.30 | -5.77133 |
|  | XP\_065062106\_8080-model2.31 | -5.75454 |
|  | XP\_065062106\_8080-model2.32 | -5.75421 |
|  | XP\_065062106\_8080-model2.33 | -5.75421 |
|  | XP\_065062106\_8080-model2.34 | -5.73246 |
|  | XP\_065062106\_8080-model2.35 | -5.73246 |
|  | XP\_065062106\_8080-model2.36 | -5.71859 |
|  | XP\_065062106\_8080-model2.37 | -5.63526 |
|  | XP\_065062106\_8080-model2.38 | -5.56456 |
|  | XP\_065062106\_8080-model2.39 | -5.55827 |
|  | XP\_065062106\_8080-model2.40 | -5.19696 |
|  | XP\_065062106\_8080-model2.41 | -4.93863 |
|  | XP\_065062106\_8080-model2.42 | -4.93863 |
|  | XP\_065062106\_8080-model2.43 | -4.72032 |
| # cluster 3 - Size: 21 |  |  |
|  | XP\_065062106\_8080-model3 | -7.08149 |
|  | XP\_065062106\_8080-model3.1 | -7.02918 |
|  | XP\_065062106\_8080-model3.2 | -7.02918 |
|  | XP\_065062106\_8080-model3.3 | -7.02444 |
|  | XP\_065062106\_8080-model3.4 | -6.90602 |
|  | XP\_065062106\_8080-model3.5 | -6.84131 |
|  | XP\_065062106\_8080-model3.6 | -6.74882 |
|  | XP\_065062106\_8080-model3.7 | -6.73744 |
|  | XP\_065062106\_8080-model3.8 | -6.70873 |
|  | XP\_065062106\_8080-model3.9 | -6.56165 |
|  | XP\_065062106\_8080-model3.10 | -6.50134 |
|  | XP\_065062106\_8080-model3.11 | -6.495 |
|  | XP\_065062106\_8080-model3.12 | -6.46177 |
|  | XP\_065062106\_8080-model3.13 | -6.42783 |
|  | XP\_065062106\_8080-model3.14 | -6.42783 |
|  | XP\_065062106\_8080-model3.15 | -6.40271 |
|  | XP\_065062106\_8080-model3.16 | -6.40271 |
|  | XP\_065062106\_8080-model3.17 | -6.31374 |
|  | XP\_065062106\_8080-model3.18 | -5.99344 |
|  | XP\_065062106\_8080-model3.19 | -5.73395 |
|  | XP\_065062106\_8080-model3.20 | -4.81547 |
| # cluster 4 - Size: 19 |  |  |
|  | XP\_065062106\_8080-model4 | -6.72185 |
|  | XP\_065062106\_8080-model4.1 | -6.27282 |
|  | XP\_065062106\_8080-model4.2 | -6.16922 |
|  | XP\_065062106\_8080-model4.3 | -6.16922 |
|  | XP\_065062106\_8080-model4.4 | -6.16922 |
|  | XP\_065062106\_8080-model4.5 | -6.16922 |
|  | XP\_065062106\_8080-model4.6 | -5.82361 |
|  | XP\_065062106\_8080-model4.7 | -5.82361 |
|  | XP\_065062106\_8080-model4.8 | -5.80067 |
|  | XP\_065062106\_8080-model4.9 | -5.80067 |
|  | XP\_065062106\_8080-model4.10 | -5.72312 |
|  | XP\_065062106\_8080-model4.11 | -5.6231 |
|  | XP\_065062106\_8080-model4.12 | -5.56774 |
|  | XP\_065062106\_8080-model4.13 | -5.44798 |
|  | XP\_065062106\_8080-model4.14 | -5.44798 |
|  | XP\_065062106\_8080-model4.15 | -5.40582 |
|  | XP\_065062106\_8080-model4.16 | -5.33076 |
|  | XP\_065062106\_8080-model4.17 | -5.29681 |
|  | XP\_065062106\_8080-model4.18 | -4.9795 |
| # cluster 5 - Size: 2 |  |  |
|  | XP\_065062106\_8080-model5 | -5.7975 |
|  | XP\_065062106\_8080-model5.1 | -4.92971 |
| # cluster 6 - Size: 7 |  |  |
|  | XP\_065062106\_8080-model6 | -5.29064 |
|  | XP\_065062106\_8080-model6.1 | -5.1046 |
|  | XP\_065062106\_8080-model6.2 | -5.07673 |
|  | XP\_065062106\_8080-model6.3 | -4.98663 |
|  | XP\_065062106\_8080-model6.4 | -4.92705 |
|  | XP\_065062106\_8080-model6.5 | -4.90263 |
|  | XP\_065062106\_8080-model6.6 | -4.87728 |
| # cluster 7 - Size: 2 |  |  |
|  | XP\_065062106\_8080-model7 | -4.75179 |
|  | XP\_065062106\_8080-model7.1 | -4.50762 |
|  |  |  |
